# Radionuclide monitoring in foodstuff: overview of the current implementation in the EU countries

**DOI:** 10.1007/s10967-014-3773-y

**Published:** 2014-11-27

**Authors:** Borbála Máté, Katarzyna Sobiech-Matura, Timotheos Altzitzoglou

**Affiliations:** European Commission, Joint Research Centre (JRC), Institute for Reference Materials and Measurements (IRMM), Standards for Nuclear Safety, Security and Safeguards Unit, Retieseweg 111, 2440 Geel, Belgium

**Keywords:** Foodstuff, Radionuclide, Monitoring, Regulation, Reference material

## Abstract

The Member States (MS) of the European Union (EU) are obliged to monitor the radioactivity in the environment since the signature of the Euratom Treaty (Treaty establishing the European Atomic Energy Community). Numerous secondary legislations derived from the Treaty can be found stating restrictions and maximum permitted levels of radionuclides in foodstuff. But to that purpose, no common integrated measurement methods are used with well-defined measurands. The present work consists of two parts. First, the current European regulations in force were collected, and then the food monitoring results, provided by the MS, were analysed.

## Introduction

Nowadays increasing numbers of citizens around the world are concerned about the levels of radioactive and other contamination in the environment arising from human activities. After the Chernobyl and Fukushima nuclear accidents it became obvious, that precise and rapid methods are necessary for routine determination of artificial radionuclide content in foodstuff [1]. On the other hand, the amount of naturally occurring radioactive material (NORM) utilised and produced by the non-nuclear industries is increasing. This creates the implicit need to deal with the waste from these activities and shows the importance of intensifying natural isotope monitoring in the food chain [2].

The analysis of the effects of previous nuclear accidents could serve in reducing the effects of any future nuclear emergencies. To improve impact assessments of radionuclide contamination, accurate information is needed on the normal levels in monitored foodstuff [3]. The Member States (MS) of the European Union (EU) have the legal obligation to monitor the radioactivity in their environment already since the signature of the Euratom Treaty (Treaty establishing the European Atomic Energy Community). The MS established their national radionuclide monitoring programmes and networks for drinking water, milk and dairy products, mixed diet and other foodstuff monitoring. This means that thousands of measurements are carried out each year. Our aim was to get an overview of the current implementation of radionuclide monitoring in foodstuff in the MS of the EU.

## The legal basis of the radionuclide monitoring in EU

Every action taken by the EU is founded on treaties. Those are binding agreements between all MS. These treaties lay down the EU objectives, the rules for EU institutions, the decision making and the relationship between the EU and its MS [4]. Derived from the treaties, the secondary legislation is the base for the regulations in the MS. The secondary legislation can be:

- Regulation: binding legislative act which must be applied immediately in every MS;
- Directive: the goal is strictly defined but every MS can decide how to achieve it;
- Decision: directly applicable by those to whom it is addressed, MS or individuals;
- Recommendation: similar to a directive, but without any legal consequences; it is an instrument of the indirect action aiming at the preparation of legislation in MS.

The three main institutions involved in creation of the EU legislation are the European Parliament (EP), the Council of the European Union (EU Council) and the European Commission (EC). By functionality the EP represents the EU citizens and is directly elected by them; the EU Council represents the governments of the individual member countries and the EC represents the interests of the Union as a whole. These institutions create the policies and laws through the ordinary legislative procedure. This means that the EC drafts and proposes the EU legislation. The role of EP and EU Council is to consider the proposals and by voting accept it or reject and send for revision. The EC and the MS then implement the laws, and the EC ensures their proper implementation and application [4].

### The Euratom Treaty

The Euratom Treaty, established in 1957, contains the primary legislation in which the radionuclide monitoring in the environment is described. The aim of the Treaty is to coordinate the MS research programmes in the field of nuclear energy. It concentrates on the most important issues related to radioactivity as promoting research, establishing uniform safety standards and facilitating investments [5].

Out of the 177 articles of the Treaty the following eight are establishing the regulatory basis of our research:

- *Articles 8 and 39* state the establishment of the Joint Nuclear Research Centre and of the Central Bureau for Nuclear Measurements (CBNM). This bureau was established in 1960 and renamed in 1993 as Institute for Reference Materials and Measurements (IRMM). This Institute is now one of the seven institutes of the Joint Research Centre (JRC) of the European Commission (EC).
- *Articles 30, 32 and 33* stipulate that basic standards should be laid down for the protection of the health of workers and the general public against the dangers arising from ionizing radiations.
- *Article 31* defines the group of experts which shall have advisory status for risk assessments, standards, proposals and opinions.
- *Article 35* says that each MS shall establish the facilities necessary to carry out continuous radioactivity monitoring and the EC shall have access to them in order to verify their operation and efficiency.
- *Article 36* describes that the EC should be informed about the level of radioactivity to which the public is exposed.

### Verification issues according to the Article 35 of the Euratom Treaty

According to the Article 35 of the Treaty, verification of the operation and efficiency of facilities, which monitor the radioactivity continuously in the environment, are carried out. The documentation of one verification visit consists of two parts: main findings and technical reports. In order to obtain the overview of the foodstuff monitoring programmes we used the technical reports as the basic documents [6]. The reports contain the detailed description of the national radioactivity monitoring programmes. The verification scheme is to carry out five to eight verifications per year in order to guarantee that approximately one audit can be conducted in each MS every five years.

In the period from 1990 to present, 85 verifications were realized as follows:

- 1990–2003, 23 verifications: in the first phase the main aim was to have an overview of the facilities monitoring the levels of radioactivity.
- 2004–2007, 25 verifications: the second phase focused on the most sensitive installations and on the new MS.
- 2008–, 37 verifications: the third phase focused on the environmental radiological monitoring of current and former uranium mining sites and milling activities and on the environmental radiological monitoring at nuclear departments of large hospitals.

### Secondary regulations related to radionuclide monitoring in the EU

At the time of the Chernobyl accident, neither standards nor authorities had been established related to radioactive contamination in foodstuff. This accident was the starting point for the development of an integrated food safety-related radiological protection system.

The regulations relevant to radionuclide monitoring in foodstuff are collected in Table 1 [7].

**Table 1:** Regulations related to the radionuclide monitoring in foodstuff [7]

**Basic safety standards**	
Council Directive (Euratom) No. 51/2013	Laying down requirements for the protection of the health of the general public with regard to radioactive substances in water intended for human consumption. OJ L 296:12–21
Council Directive (Euratom) No. 59/2013	Laying down basic safety standards for protection against the dangers arising from exposure to ionizing radiation, and repealing Directives 89/618/Euratom, 90/641/Euratom, 96/29/Euratom, 97/43/Euratom and 2003/122/Euratom. OJ L 13:1–73
**Environmental and foodstuff monitoring**	
Commission Recommendation (Euratom) No. 473/2000	On the application of Article 36 of the Euratom Treaty concerning the monitoring of the levels of radioactivity in the environment for the purpose of assessing the exposure of the population as a whole. OJ L 191:37–46
**Maximum permitted levels in foodstuff after emergency exposure**	
Council Regulation (Euratom) No. 3954/87	Laying down maximum permitted levels of radioactive contamination of foodstuffs and of feedingstuffs following a nuclear accident or any other case of radiological emergency. OJ L 371:11–13
Commission Regulation (Euratom) No. 944/89	Laying down maximum permitted levels of radioactive contamination in minor foodstuffs following a nuclear accident or any other case of radiological emergency. OJ L 101:17–18
Council Regulation (Euratom) No. 2218/89	Amending Regulation (Euratom) No. 3954/87 laying down maximum permitted levels of radioactive contamination of foodstuffs and of feedingstuffs following a nuclear accident or any other case of radiological emergency. OJ L 211:1–3
Council Regulation (EEC) No. 2219/89	On the special conditions for exporting foodstuffs and feedingstuffs following a nuclear accident or any other case of radiological emergency. OJ L 211: 4–5
Commission Regulation (Euratom) No. 770/90	Laying down maximum permitted levels of radioactive contamination of feedingstuffs following a nuclear accident or any other case of radiological emergency. OJ L 83:78–79
**Chernobyl affected areas**	
Council Regulation (EEC) No. 1707/86	On the conditions governing imports of agricultural products originating in third countries following the accident at the Chernobyl nuclear power-station. OJ L 146:88–90
Council Regulation (EEC) No. 737/90	On the conditions governing imports of agricultural products originating in third countries following the accident at the Chernobyl nuclear power-station. OJ L 82:1–6
Council Regulation (EC) No. 616/2000	Amending Regulation (EEC) No. 737/90 on the conditions governing imports of agricultural products originating in third countries following the accident at the Chernobyl nuclear power station. OJ L 75:1–2
Commission Regulation (EC) No. 1609/2000	Establishing a list of products excluded from the application of Council Regulation (EEC) No 737/90 on the conditions governing imports of agricultural products originating in third countries following the accident at the Chernobyl nuclear power station. OJ L 185:27–29
Commission Recommendation (EC) No. 274/2003	On the protection and information of the public with regard to exposure resulting from the continued radioactive caesium contamination of certain wild food products as a consequence of the accident at the Chernobyl nuclear power station. OJ L 99:55–56
Commission Regulation (EC) No. 1635/2006	Laying down detailed rules for the application of Council Regulation (EEC) No. 737/90 on the conditions governing imports of agricultural products originating in third countries following the accident at the Chernobyl nuclear power-station. OJ L 306:3–9
Council Regulation (EC) No. 733/2008	On the conditions governing imports of agricultural products originating in third countries following the accident at the Chernobyl nuclear power station. OJ L 201:1–7
Council Regulation (EC) No. 1048/2009	Amending Regulation (EC) No 733/2008 on the conditions governing imports of agricultural products originating in third countries following the accident at the Chernobyl nuclear power station. OJ L 290:4–4
**Import of foodstuff from Japan after Fukushima nuclear accident**	
Commission Implementing Regulation (EU) No. 996/2012	Imposing special conditions governing the import of feed and food originating in or consigned from Japan following the accident at the Fukushima nuclear power station and repealing Implementing Regulation (EU) No 284/2012. OJ L 299:31–41
Commission Implementing Regulation (EU) No. 495/2013	Amending Implementing Regulation (EU) No. 996/2012 imposing special conditions governing the import of feed and food originating in or consigned from Japan following the accident at the Fukushima nuclear power station. OJ L 143:3–10
Commission Implementing Regulation (EU) No. 322/2014	Imposing special conditions governing the import of feed and food originating in or consigned from Japan following the accident at the Fukushima nuclear power station. OJ L 95:1–11

The Commission Recommendation (Euratom) No. 473/2000 [8] is the regulatory base of the radionuclide monitoring in foodstuff. It stipulates that not only environmental samples, but also food samples should be monitored, in order to assess the exposure of the population as a whole. Additionally, according to this recommendation, sparse and dense networks should be set up:

- Sparse network is a monitoring network comprising for every region and for every sampling medium at least one location representative of that region. At such locations high sensitivity measurements should be performed thus giving a transparent representation of actual levels as well as about their trends.
- Dense network is a monitoring network comprising sampling locations distributed throughout the MS’s territory in such a way that allows the EC to compute regional averages for radioactivity levels in the community.

## Evaluation of the collected data

In this chapter the main critical findings from the regulations and from the verification issues are collected. According to the verification documents, the type of sample, the sampling frequency and the measurand (radioisotopes) were sorted by countries.

### Implementation of international regulations

The implementation of international regulations to the national legislation is not fully harmonized. For example in the technical documents 20 different terms are used to designate activity limits (detection, standard, temporary, pre-set, alarm, exposure, threshold, permissible, maximum, quantification, release, derived release, operational release, operational, discharge, emission, regulatory, regulatory discharge, authorised and decision limit) and 28 different terms are used to designate activity levels (alarm, pre-set alarm, alert, emergency, action, recommended action, intervention, warning, fixed (radiation alarm), permissible, maximum permitted, threshold, maximum, accepted, decision, authorised, production, exemption, optimization, release (accepted), clearance, guidance, reference, diagnostic reference, monitoring, controlling, screening and reporting level). This could be partly originating from the interpretation of the secondary legislation [9].

Listed remarks in connection to the secondary legislation in EU are the following:

- No restrictions can be found for normal conditions, only for emergency situations,
- No authority is dedicated, which could immediately react in the case of non-compliance or emergency,
- Guidelines and restrictions are established for processed food and agricultural products not for raw material or concentrated or dried food,
- Important details of sample preparation are not precisely determined (as washed, skinned, peeled).

### Definition of the sample

Sample types can be divided into four groups: drinking water, milk and milk products, mixed diet and other foodstuff samples. The definition of the sample is not precisely given, however it is obvious that the sample representativeness is one of the most important issues in any kind of environmental monitoring [10].

The most significant difference between the MS monitoring programmes is the definition of the mixed diet sample. It can be defined by the national authority as a typical daily diet (complete meals) or the separate ingredients of the meals, representing the consumption of the whole population of the MS. It can be also even not in the monitoring programme as mixed diet, but only as separated elements listed in other foodstuff category. The typical daily diet can contain a complete meal consisting of one dish or of all food produced in a canteen/hospital within 24 h. In some cases even the solid and liquid foodstuff originating from one 24-h sampling are analysed separately.

In connection to the other foodstuff samples several MS collect it randomly from local markets, which means that they collect available vegetables, fruits and meat samples without any predefined schedule based only on availability. However in other cases not only the collecting place, but also the species and the amount of samples are strictly regulated; for example 10 kg carrots, 5 kg cabbages, 6 kg potatoes and so on have to be collected annually.

### Foodstuff sampling programmes and sampling frequency

Generally it can be concluded that the sampling systems are not fully harmonised as well, only a few MS are applying the sparse and dense network scheme. In some MS the monitoring activities are concentrated in the vicinity of special facilities and are not covering the whole country. Additionally, in a few MS the monitoring programmes are even coordinated by regional authorities following different schemes. The sampling frequencies for the different samples can be found in Table 2. Drinking water is monitored mostly on monthly basis. Milk and milk product samples are taken weekly/monthly and monthly/quarterly samples are created from the smaller samples. According to Table 2 no correlation can be found in mixed diet sampling frequency of the MS, while other foodstuff samples are monitored mainly annually.

**Table 2:** Sampling frequency in the EU MS for foodstuff monitoring. The numbers represent the number of MS taking the given foodstuff sample with the specified frequency

Sampling frequency	Sample type			
	Drinking water	Milk and milk products	Mixed diet	Other foodstuff
Monthly	11	9	6	2
Quarterly	4	10	4	2
Twice/year	3	1	5	2
Annually	3	0	4	10
Other	3	4	0	5
n.i.^a^/n.a.^b^	4	4	9	7
EU MS (total)	28	28	28	28

^a^ *n.i.* no information

^b^ *n.a.* not applicable

### Measurands and measurement methods applied

In the MS radioactivity is measured by national dedicated monitoring laboratories in different environmental and food samples. In most of the cases the laboratories are accredited and/or certified for methods they use. But still Europe-wide different measurement methods are applied to determine the same nuclides, sometimes without well-established quality control tools.

Determination of ^90^Sr and gamma-ray emitting radionuclides (^137^Cs, ^40^K) are carried out almost in every type of samples. In the case of drinking water ^3^H and gross alpha/beta are measured additionally. The radionuclides measured in the food samples are collected in Table 3.

**Table 3:** Measurands in foodstuff samples in EU MS monitoring programmes. The numbers represent the number of MS who monitor the specified measurand in the foodstuff sample

Measurand	Sample type			
	Drinking water	Milk and milk products	Mixed diet	Other foodstuff
Gross alpha/beta	16	4	2	5
Gamma-ray emitters	17	25	17	22
^90^Sr	18	24	18	15
^3^H	22	1	0	2
Natural isotopes	9	3	3	6

The measurements of the caesium and potassium isotopes are one of the simplest via gamma-ray spectrometry method; most of the laboratories can determine those radioisotopes without any problems. Difficulties could occur when complex chemical separation (^90^Sr) [11] or complicated calculation method should be applied [12].

The gross methods are in many cases not as simple as they are described. Very significant discrepancies were obtained between the results of national monitoring laboratories measurement performance. It can be partly explained by the lack of knowledge of the real radionuclide composition. According to Jobbagy et al. [13] the method should be applied only in cases where in the radiochemical composition of the sample no temporary change is expected, no complex decay chains can be found in the sample and a true standardized method is applied with fixed measurement parameters. Total or gross activity measurements are of limited value for determining actual radioactivity content however they are still among the most frequently used ones. On the other hand it is important to mention that these methods under controlled conditions provide important information in trend analysis and are sensitive to detecting changes in such trends.

Only some countries are monitoring both the artificial radionuclides and the natural occurring radioisotopes radionuclide-specifically. Mostly ^226^Ra and in some cases also U and ^210^Po isotopes are examined.

## Conclusion

As it was shown, numerous secondary legislations can be found across the EU which state the restrictions, guidelines and maximum permitted levels of radionuclides in foodstuff. But, there are no commonly used integrated measurement methods with well-defined measurands and calculation methods. Several laboratories still use in-house developed, but not accredited and/or well controlled methods for the measurements.

One possibility to improve the monitoring activities of the MS is the development of the performance of laboratories in radioactivity measurement. That means the improvement of the measurement conditions and the level of expertise. This can be carried out via inter-laboratory comparisons, where the underperforming laboratories can be identified [14]. With a validated and well controlled measurement method, advice can be given to them on how to improve or modify their methods. On the other hand providing certified food reference material helps to improve the comparativeness of the measured results.

JRC-IRMM has the responsibility to organise the EC inter-laboratory comparisons since 2003. Foodstuff samples as milk powder, drinking water and bilberry powder were already used. The bilberry material is one of the newest certified reference materials (IRMM-426), which can be used for the validation of radionuclide measurement methods at the monitoring laboratories [15]. The aim of the inter-laboratory comparisons is not only to evaluate the results obtained from the laboratories, but also to help them to improve their measurements and methods via workshops and meetings.

Our plan for the future is to establish an inventory of food measurement methods. Since important information dealing with the radionuclide monitoring in foodstuff cannot be found in the technical documents, a questionnaire is prepared and will be sent soon to the national representatives of the MS. It contains thematic questions concerning the monitored foodstuff and details of monitoring programmes and the measurement methods of radionuclides in foodstuff. A question is also included about the current and future needs of the laboratories of food reference materials.

## Appendix

### Word-by-word quotations

Definition of Sparse and Dense network from the Commission Recommendation (EURATOM) No. 473/2000:
Sparse network is a monitoring network comprising for every region and for every sampling medium at least one location representative of that region. At such locations high sensitivity measurements should be performed thus giving a transparent representation of actual levels as well as about their trends. Dense network is a monitoring network comprising sampling locations distributed throughout the MS’s territory in such a way that allows the EC to compute regional averages for radioactivity levels in the community.
*Reference:* Commission Recommendation (Euratom) No. 473/2000 of 8 June 2000 on the application of Article 36 of the Euratom Treaty concerning the monitoring of the levels of radioactivity in the environment for the purpose of assessing the exposure of the population as a whole. OJ L 191: 37–64.

Definition of basic standards according to the Euratom Treaty:
Basic standards should be laid down for the protection of the health of workers and the general public against the dangers arising from ionizing radiations.
*Reference:* Consolidated version of the Treaty establishing the European Atomic Energy Community (2012) OJ C 327:1–107.
